# Co-Circulation of Divergent Strains Supports Vector-Mediated Transmission of Rodent Hepacivirus J (*Orthohepacivirus glareoli*)

**DOI:** 10.3390/v18060651

**Published:** 2026-06-05

**Authors:** Sarah Marmorosch, Thomas Anton von Graffenried, Rainer G. Ulrich, Gerald Heckel

**Affiliations:** 1 Institute of Ecology and Evolution, University of Bern, 3012 Bern, Switzerland; 2 Institute of Novel and Emerging Infectious Diseases, Friedrich-Loeffler-Institut, Federal Research Institute for Animal Health, 17493 Greifswald-Insel Riems, Germany; rainer.ulrich.gast@fli.de

**Keywords:** RNA virus, *Hepaciviridae*, rodent hepacivirus, bank vole-associated hepacivirus, Rodent Hepacivirus J, *Orthohepacivirus glareoli*, vector-borne transmission

## Abstract

*Orthohepacivirus glareoli* (RHVJ, *Hepaciviridae*) and its natural host, the bank vole (*Myodes glareolus*), have been proposed as a model system for human hepatitis C research, yet the mode of transmission remains largely unknown. Here, we investigated spatial patterns of RHVJ sequence diversity and evolutionary relationships using novel sequences from dense regional sampling alongside all published NS3 gene sequences. Phylogenetic analyses revealed mostly local clustering of RHVJ sequences, resulting in isolation-by-distance patterns at regional geographic scales. This suggests primarily local transmission of the virus. However, nucleotide sequence divergence of up to 19% within bank vole populations is difficult to reconcile with local transmission of RHVJ alone, implying that mechanisms beyond local evolution shape the extensive sequence diversity of RHVJ at local scales. Using spatially explicit computer simulations of sequence evolution, we contrasted the phylogenetic relationships resulting from exclusively short-distance transmission, e.g., from vole to vole, with those resulting when rare long-distance transmission events were included. The latter produced phylogenetic patterns comparable to those of RHVJ, including monophyletic clustering of samples from distant locations and unresolved basal nodes. We suggest that the transmission of RHVJ likely involves at least occasionally a vector, as the mobility of its natural rodent host is strongly limited.

## 1. Introduction

Hepaciviruses (family *Hepaciviridae*) are emerging pathogens, some of which are of great importance for global public health [1]. Their unsegmented genome of ~10 kilobases (kb) is composed of a positive-sense single-stranded RNA molecule and encodes a single polyprotein that is processed into three structural and seven nonstructural (NS) proteins [1,2]. There are currently 14 distinct hepacivirus species recognized, among which the most studied species is the human hepatitis C virus (HCV), classified as *Orthohepacivirus hominis*, one of the leading causes of chronic liver disease in humans [1,3,4].

The bank vole (*Myodes glareolus*, syn. *Clethrionomys glareolus*) is a widely distributed forest-dwelling rodent species across Europe [5]. It is a reservoir host for a variety of viruses, including Puumala virus (PUUV), an orthohantavirus, and Tick-borne encephalitis virus (TBEV), which belongs to the orthoflaviviruses [1,6,7,8]. Additionally, two novel hepacivirus species have been discovered in bank voles, namely rodent hepacivirus J (RHVJ) and rodent hepacivirus F (RHVF), which were later classified as *Orthohepacivirus glareoli* and *Orthohepacivirus myodae* [1,9,10]. A survey of various small mammals revealed a very strong association of both viruses with bank voles and a wide geographic spread across Central and Northern Europe [11].

Experimental infection of bank voles with these bank vole-associated hepaciviruses (BvHV) has revealed pathological signs comparable to those observed during human HCV infection, including liver tropism and chronic hepatitis, and has thus been proposed as a model system for studying early stages of HCV infection in humans [12]. However, unlike HCV, which is primarily transmitted through blood exposure such as blood transfusion and intravenous drug use, the main transmission mode of BvHV remains unknown, although BvHV RNA has been detected in blood [9,12]. Direct transmission among bank voles or vertical transmission, for example, have been described as inefficient or absent, while alternative mechanisms such as vector-borne transmission have been proposed [12]. Vector-mediated transmission has also been suggested for several other hepaciviruses, based on their detection in blood-feeding arthropods that were collected from or near their vertebrate host [13,14,15]. However, direct evidence for the involvement of an arthropod vector for BvHV transmission is lacking [11].

In this study, we focus on RHVJ which appears to be more abundant in Central Europe than RHVF [11]. Despite its relatively high prevalence in natural bank vole populations, patterns of viral circulation remain largely unknown [11]. Spatial patterns of viral diversity are closely linked to the transmission mechanisms and are therefore often exploited to infer viral circulation within and among host populations. Viruses that rely exclusively on short-distance transmission (i.e., from vole to vole) in natural rodent populations without human association spread primarily at local scales, due to the restricted movement of their hosts [16,17,18]. Such local circulation of viral populations typically results in phylogenetic relationships characterized by local sequence clusters and isolation-by-distance patterns at regional geographic scales, as seen for instance in vole-associated hantaviruses [16,19,20,21]. In contrast, viruses that circulate in highly mobile hosts or involve vector species in their spread can experience occasional long-distance transmission, leading to geographically mixed phylogenetic trees and weak or no association between genetic and geographic distance [22]. Such spatial patterns of viral diversity have been demonstrated for example for globally circulating human influenza viruses and severe acute respiratory syndrome (SARS)-related coronaviruses and are commonly exploited to reconstruct transmission histories during outbreaks in humans and livestock [22,23].

In this study, we assess spatial patterns of RHVJ diversity and reconstruct evolutionary relationships across Europe. To gain more insights into the transmission dynamics that drive viral circulation in natural bank vole populations, we use dedicated population sampling at the regional scale in Switzerland. Furthermore, we use spatially explicit computer simulations to contrast the effects of only local circulation of virus strains versus occasional long-distance transmission in phylogenetic trees.

## 2. Materials and Methods

### 2.1. Bank Vole Collection in Switzerland

Bank voles were sampled at 14 different locations across central and western Switzerland between the years 2015 and 2023 (Table 1 and Table S1). Voles were captured using snap traps and stored at −80 °C after collection. Lung and liver samples were collected and subsequently stored at −80 °C.

### 2.2. RNA Extraction and RT-PCR Analyses

RNA was extracted from either lung or liver tissue using a standard QIAzol protocol. All samples were screened with RT-PCR using the SuperScript III One-Step RT-PCR System with Platinum Taq DNA Polymerase (Invitrogen, Thermo Fisher Scientific, Waltham, MA, USA). Reverse transcription and amplification involved 45 min at 50 °C, 2 min at 95 °C, 40 cycles of 30 s at 94 °C, 30 s at 52 °C, 1 min at 68 °C, a final extension of 10 min at 68 °C and cooling at 4 °C. PCR amplification was performed using the primers rodHV_NS3_932F (5′- CGG AAA GAC CAC YAA RGT GCC-3′) and rodHV_NS3_1567R (5′- AAG TTT CCD GTG TAD CCV GTC AT-3′), targeting a fragment of the NS3 gene [11]. This fragment codes for the helicase domain of the nonstructural protein 3 (NS3), a conserved region that is essential for viral replication [24,25]. RT-PCR products were analyzed with gel electrophoresis (1.5% agarose gel, stained with GelRed Nucleic Acid Gel Stain (Biotium, Fremont, CA, USA)), by comparing band sizes to a positive control (previously sequenced sample MglCHBkB03). PCR products of the expected size were cleaned using ExoSAP-IT PCR Product Cleanup Reagent (Applied Biosystems, Thermo Fisher Scientific, Waltham, MA, USA) and sequenced with the dideoxy chain termination method by Microsynth (Microsynth AG, Balgach, Switzerland). The hepacivirus species was identified using Basic Local Alignment Search Tool (BLAST v 2.17.0), based on the highest sequence similarity and coverage with reference sequences in GenBank [26]. The number of distinct sequence types (i.e., unique sequences) was determined for all RHVJ sequences from Switzerland using DnaSP version 6.12.03 [27].

### 2.3. Genetic Divergence and Isolation-by-Distance Analyses

Pairwise genetic divergence between RHVJ sequences was calculated in R version 4.4.3 as p-distance and the sum of branch lengths for both nucleotide and amino acid sequences using the *ape* package version 5.8-1 [28,29]. Both transitions and transversions were considered and pairwise deletion was defined for alignment gaps. The sum of branch lengths between samples was derived from the Bayesian phylogenetic analyses described below. To assess genetic variability at local scales, we calculated mean within-population genetic distances in R. Distributions of within-population genetic distance for locations with at least four available sequences were plotted in R using the *ggplot2* package, including the standard deviation [30]. We tested for an overall association between genetic and geographic distance across all RHVJ sequences using a Mantel test implemented in the *vegan* package version 2.7-1 [31]. For investigating scale-dependent correlations between genetic and geographic distance, we used a Mantel correlogram with distance classes of 200 km intervals (up to 1600 km). We applied a cutoff that limited the correlogram to the distance classes that contained all samples, resulting in Mantel correlation coefficients for distance classes up to 800 km. Geographic distances between sampling locations were computed with the *geodist* package version 0.1.1 [32]. Samples collected from the same host population were treated as sharing identical coordinates of origin and were therefore assigned a geographic distance of zero. In addition, we performed a full exploratory scan of recombination in RDP5 (v5.84) using the RDP, GENECONV, BootScan, MaxChi, Chimaera, SiScan, and 3Seq methods [33]. We applied a significance threshold of *p* < 0.05 and used the default parameter values for each method.

To assess evolutionary pressures acting on the NS3 gene, we calculated the ratio of nucleotide diversity at non-synonymous and synonymous sites (πa/πs) in DnaSP with the Jukes and Cantor correction applied [34]. Sliding window analysis was performed with a window length of 30 and a step size of 10 nucleotides along the partial NS3 gene sequences. The datasets obtained from the sliding window analyses were imported into R and visualized using the *ggplot2* package.

### 2.4. Phylogenetic Reconstruction

The phylogenetic reconstruction included our newly generated sequences and all publicly available sequences of RHVJ strains (Table S2). Newly generated sequences were trimmed to the length of publicly available sequences, resulting in a final alignment of 108 RHVJ sequences with a length of 468 nucleotides (nt) each. One RHVF sequence from north-east Germany (GenBank: MW822242) was used as the outgroup [11]. The sequences were aligned using ClustalW Multiple Alignment in BioEdit version 7.7.1 [35]. MrBayes v. 3.2.7 was used for the phylogenetic reconstruction [36]. Model selection was performed with the ModelFinder analysis in the web server of IQ-Tree [37]. The best-fit substitution model based on the Bayesian information criterion (BIC) scores was a General Time Reversible (GTR) substitution model with gamma-distributed rate variation among sites and a proportion of invariable sites (GTR + I + Γ). Two independent runs of Markov chain Monte Carlo (MCMC) analysis were performed for up to 10^7^ generations. An automatic stopping rule was implemented and set to a standard deviation of split frequencies of 0.01. Sampling was conducted every 1000 generations with a burn-in value set to 0.25. The resulting tree was visualized in iTOL and nodes with posterior probability values ≤ 0.8 were removed [38].

### 2.5. Spatially Explicit Simulations of Viral Evolution

To determine whether the observed phylogenetic patterns could be associated with occasional long-distance viral transmission, we used computer simulations for two transmission scenarios as a proof of principle. We simulated the evolution of a non-recombining RHVJ population in a continuous two-dimensional space using a custom R script with predefined random seeds for the simulations (see https://github.com/thomasvongraffenried/RHVJ-Simulations, accessed on 1 May 2026). The simulation space was modelled as a two-dimensional square ranging from 0 to 1 in both x and y coordinates. Each simulation started with a single sample in the center of the landscape (x = 0.5, y = 0.5), consisting of a 468 nt-long sequence. This first sequence corresponded to the empirical sample “MglCHBkB03” from a Swiss bank vole population (Table S2). The simulated samples replicated clonally with a probability of 0.8, modelled as a Bernoulli trial. Replicating samples were set to generate up to five progenies, with one placed at the coordinates of the parental virus and the remaining progenies were transmitted along the x and y axes. Distances of viral transmission along the x and y axes were drawn from a half-normal distribution. In simulations with only local circulation, 100% of progenies followed a short-range kernel with standard deviation of σ = 5 rc, with rc representing the spatial scale parameter. To capture rare long-distance transmission events, a second simulation framework was used in which 99% of progenies followed a short-range kernel with standard deviation of σ = 5 rc, while in 1% a long-distance kernel with σ = 200 rc was used. Although each replicating sample could generate up to five progenies, the realized number was constrained by local density, the carrying capacity, and the exclusion of samples outside the simulated space. The carrying capacity restricted the entire simulated space to K = 1000 samples for each generation, while the local density parameter enforced a minimum spacing of 3 rc between new progenies to avoid dense local accumulation within the simulation space. Thus, if the number of samples exceeded K, uniform random sampling was used to discard all but 1000 samples. Furthermore, any potential progenies whose coordinates were set to be within 3 rc of previously transmitted progenies or outside of the simulated space were discarded. Progenies and their coordinates were generated sequentially. We used a site-independent mutation model with a mutation rate of 10^−3^, which is based on previous findings in the closely related HCV [39]. Both transmission scenarios were each run for up to 300 generations. The simulated sequences were exported in FASTA format using the R package *seqinr* version 4.2-36 [40].

### 2.6. Sampling of the Simulated Data and Phylogenetic Analyses

To compare the simulated output with our empirical data, we sampled sequences from two areas in the simulation space and performed phylogenetic analyses on them. The sampling areas were located in opposite corners of the simulated space and each occupied an area of 1/3 × 1/3 (Figure S1). For every generation in both transmission scenarios, we examined whether these areas contained at least 25 samples each. From the first generation when this criterion was fulfilled, we selected 25 samples per area using uniform random sampling without replacement, ensuring equal selection probability for all individuals within an area. We repeated the simulations of both transmission scenarios until we had 20 runs each. These 20 pairs were analyzed using the same phylogenetic methods applied to the real sequence data. For each phylogeny, the ancestral sequence “MglCHBkB03” was used as the outgroup.

## 3. Results

### 3.1. Detection of RHVJ in Switzerland

**Figure 1:**
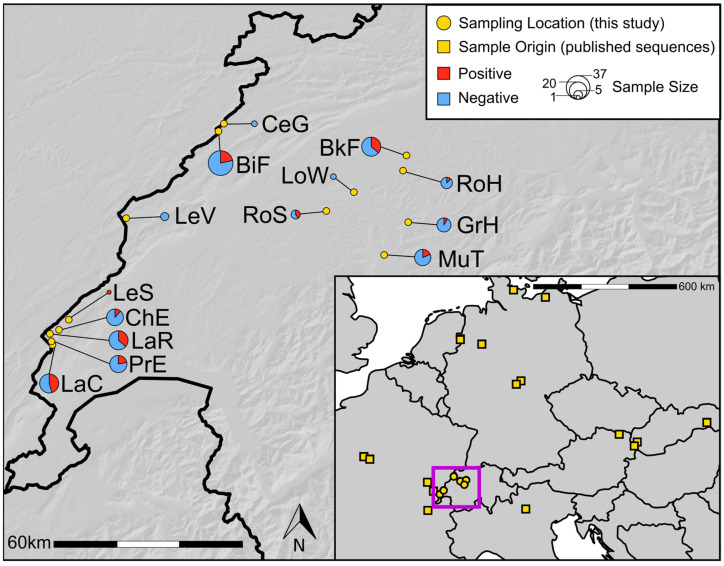
Geographic distribution and screening results of RHVJ RNA in bank voles (*Myodes glareolus*). Yellow circles represent the sampling locations of bank voles. Pie charts show the proportion of RHVJ RNA-positive (red) and RHVJ RNA-negative (blue) individuals, with size corresponding to the number of voles tested. Different shades of grey illustrate variation in elevation. For location ID and exact sample sizes, see Table 1. The inset map displays the geographic origins of published RHVJ sequences (yellow squares) and shows the position of the Swiss sampling locations within Central Europe (purple square, Table S1).

RHVJ RNA was detected in bank voles from 11 out of 14 sampling locations across central and western Switzerland (Figure 1). It was not found at three sampling locations where sample sizes were four or fewer voles. In total, 48 out of 188 bank voles tested RT-PCR-positive for RHVJ (Table 1), resulting in an overall prevalence of 25.5% (95% confidence interval (CI): 19.8–32.2%). The highest site prevalence was found in La Cure, where 10 out of 22 bank voles (45.5%; 95% CI: 26.9–65.3%) tested positive. Repeated detection of RHVJ RNA across multiple years revealed the continued presence of RHVJ within several bank vole populations (Table S1). From the RT-PCR-positive samples, a 528 nt long fragment of the NS3 gene was sequenced from 46 samples (GenBank accession numbers: PZ404792-PZ404837). Several sequencing attempts of two samples (one each from La Cure and La Racine) failed due to strong noise signal in the electropherogram of parts of the sequence. As both samples originate from populations that were already well represented in our dataset, we excluded these samples from downstream analyses. In total, 35 distinct nucleotide sequence types were identified among the 46 RHVJ sequences from Switzerland (Table S1).

### 3.2. Spatial Patterns of Genetic Variability

Overall, sequence pairs across Europe had a mean p-distance of 15.16% at the nucleotide level. The mean genetic distance of sequences from Switzerland only was 14.83% (maximum: 21.84%), which is comparable to the mean genetic distance calculated across all other countries (excluding Switzerland; mean 14.61%, maximum 20.94%). Despite large nucleotide-level divergence in RHVJ, amino acid sequence divergence remained comparatively low, with a mean of 2.19% (maximum 5.23%) in Switzerland and 1.36% across Europe (excluding Switzerland; maximum 7.05%). Sliding window analysis of πa/πs along the partial NS3 gene sequences revealed consistently low levels of non-synonymous to synonymous nucleotide diversity with an average of 0.02 in Switzerland and 0.01 across all other countries (Figure S2).

**Figure 2:**
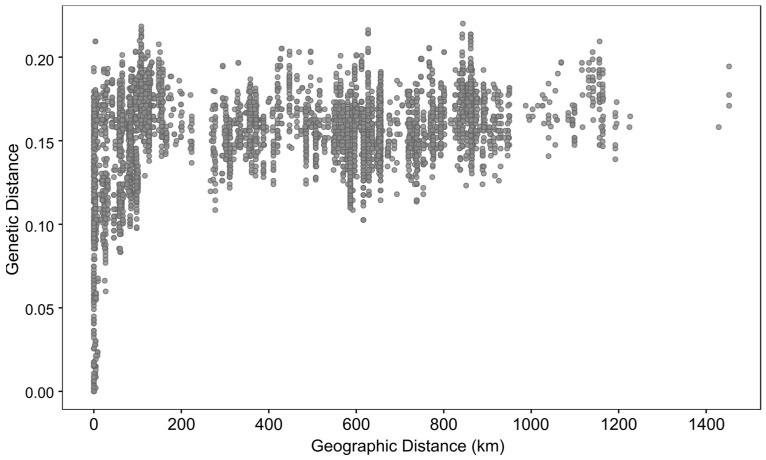
Relationship between geographic and genetic distance among partial NS3 gene sequences of RHVJ strains from Europe. Each point represents a pairwise comparison. The association is overall positive (Mantel test, r = 0.333, *p* ≤ 0.001) despite a largely flat relationship for distances > 200 km.

**Figure 3:**
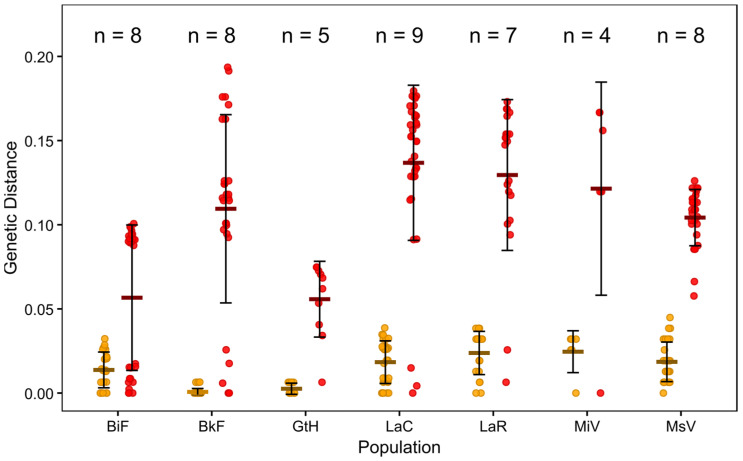
Genetic distances between RHVJ sequences obtained within bank vole populations. Each point represents a pairwise comparison between nucleotide sequences (red) and amino acid sequences (orange). Horizontal bars and whiskers indicate the mean genetic distance and standard deviation for each sequence type and location. Locations were included only if the sample size was at least four. The sample size for each location is given. BiF = Biaufond (CHE), BkF = Bütikofen (CHE), GtH = Gotha (GER), LaC = La Cure (CHE), LaR = La Racine (CHE), MiV = Mignovillard (FRA), MsV = Mont-sous-Vaudrey (FRA).

Isolation-by-distance analyses revealed overall a positive association between geographic and genetic distance, which leveled off at a geographic distance of approximately 200 km (Figure 2). Mantel tests supported the overall relationship between geographic and genetic patterns for pairwise p-distances (r = 0.333, *p* ≤ 0.001) and sum of branch lengths (r = 0.312, *p* ≤ 0.001). However, assessing scale-dependent correlations revealed that the isolation-by-distance pattern was restricted to the shortest distance class (0–200 km), whereas Mantel correlations at larger distance classes were negative and not significant (Figure S3). Despite the positive Mantel correlation within 200 km, pairwise genetic distances at a geographic distance of zero (i.e., within bank vole populations) showed substantial variation and reached levels comparable to the genetic distances observed between RHVJ samples separated by, e.g., more than 1000 km (Figure 2). Focusing our analyses on locations with a minimum of four sequences (n = 7; four Swiss, two German and one French location) revealed within-population p-distances ranging from 0 to 19% at the nucleotide sequence level and 0 to 4% at the amino acid sequence level (Figure 3; Table S1). We found no evidence of recombination among RHVJ sequences using any of the methods available in RDP5.

### 3.3. Evolutionary Relationships in RHVJ Strains

**Figure 4:**
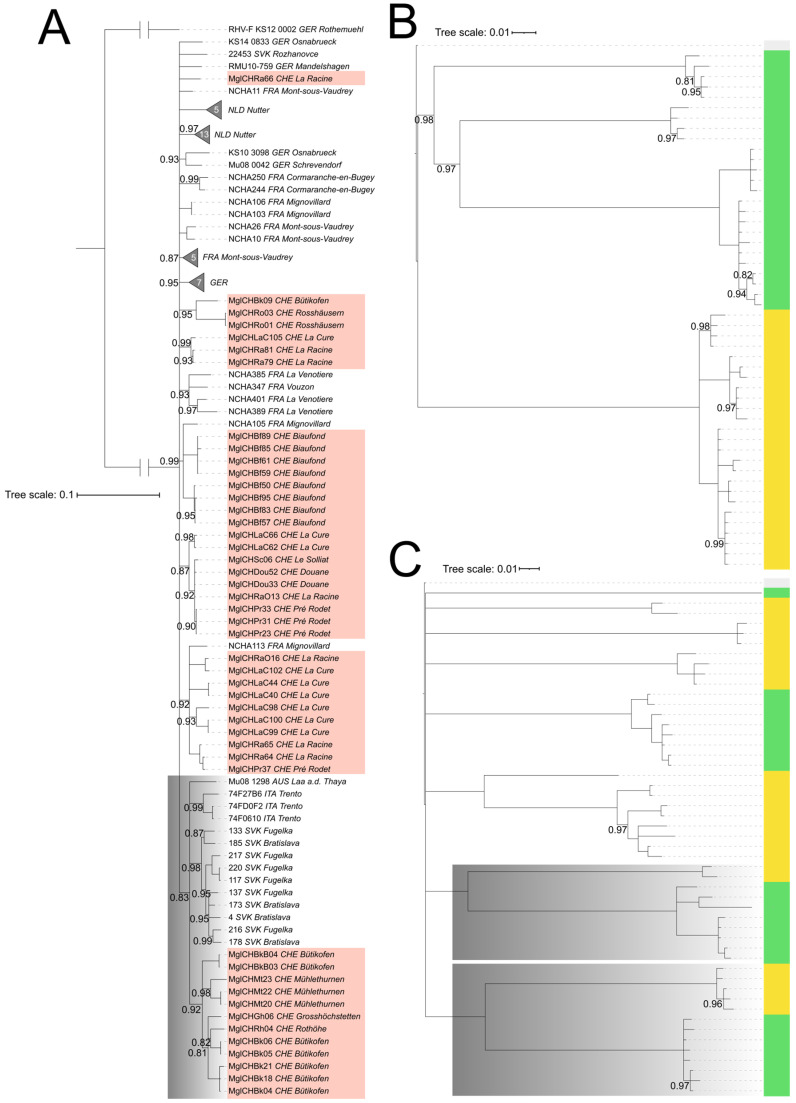
Bayesian phylogenies of *Orthohepacivirus glareoli* (RHVJ) strains based on partial sequences (468 nt) of the NS3 gene. Grey shading highlights monophyletic groups containing samples from geographically distant locations. Posterior probabilities of Bayesian inference values are shown when <1. Nodes with support of ≤0.8 were removed. Scale bars indicate evolutionary distance in substitutions per nucleotide. (**A**) Novel sequences from Switzerland (red) together with published RHVJ sequences from other regions of Europe. Sequence labels contain the three-letter code of the country of origin and the sampling location in italics. A sequence of *Orthohepacivirus myodae* (RHVF) was used as outgroup. Several clades containing sequences from the same or geographically close locations were collapsed into grey triangles for visualization purposes. The full phylogeny without collapsed clades is shown in Figure S4. (**B**,**C**) Example phylogenies obtained from spatially explicit simulations of RHVJ evolution based on 50 simulated sequences of 468 nt each (see text). Green and yellow indicate the area in the simulated space from which the sequences were sampled, corresponding to Figure S1. (**B**) Simulation scenario with local transmission of the virus only. (**C**) Scenario with local transmission and occasional long-distance transmission (1%) of the virus.

The novel sequences from Switzerland were combined with all available RHVJ sequences for phylogenetic reconstruction based on a 468 nt long fragment. Basal nodes in the phylogeny were not resolved or had relatively low support values, resulting in numerous polytomies. Nevertheless, the phylogenetic tree showed a general pattern of clustering of many sequences from the same bank vole population or geographic region, supported by high posterior probabilities of Bayesian inference values (Figure 4A). Most of the sequences from central Switzerland, for example, formed distinct monophyletic clusters in the phylogeny, suggesting local circulation of RHVJ in these populations. However, some sequences from the same location were phylogenetically divergent and clustered with sequences from distant locations. This pattern was present in the novel sequences from Switzerland (e.g., Bütikofen, La Racine) as well as in sequences from other locations across the Central European range of RHVJ (e.g., Mignovillard (FRA), Mont-sous-Vaudrey (FRA)).

### 3.4. Phylogenetic Consequences of Simulated Short and Long-Distance Transmission

Given local sequence diversity and phylogenetic patterns that were at odds with strictly local evolution of RHVJ, we used proof-of-principle computer simulations to explore potential transmission scenarios. We ran a total of 27 simulations of both short-distance and occasional long-distance transmission scenarios until 20 independent runs each were available. Seven simulations of only local circulation did not meet the specified criterion of sufficient spread after 300 generations and were thus not included in downstream analyses. We also discarded the seven simulations of occasional vector-mediated long-distance transmission with the corresponding seed to ensure an equal number of simulations per scenario.

Phylogenetic trees resulting from exclusively short-distance transmission showed distinct clusters of all samples from the same area in the simulation space (Figure 4B). Most phylogenetic trees exhibited only dichotomous or trichotomous basal nodes, and polytomies involving four or more lineages were observed only in two phylogenetic trees from the short-distance transmission scenario. For the long-distance transmission scenario, 19 out of 20 phylogenetic trees had polytomies at the basal nodes (Figure 4C). Thirteen phylogenetic trees inferred under the long-distance transmission scenario contained monophyletic clusters of samples from different sampling areas (Figure 4C), closely resembling the patterns in the empirical RHVJ phylogeny (Figure 4A). For visualization in Figure 4B,C, we selected one phylogenetic tree each that showed the typical patterns observed for each transmission scenario.

## 4. Discussion

We identified RHVJ infections in 48 bank voles from eleven different locations, demonstrating a wide distribution of RHVJ across central and western Switzerland (Figure 1). As previous studies did not include Swiss bank voles in RHVJ screening efforts, this represents the first report of RHVJ in Switzerland. The overall prevalence of 26% in Switzerland is higher than previous estimates of approximately 18% for bank vole populations across Europe, and suggests that RHVJ is a common component of the bank vole’s virome [11]. Despite the much smaller geographic scale, nucleotide-level diversity of Swiss RHVJ sequences is similar to the diversity across Europe (mean p-distance in both: 15%). This level of sequence diversity is comparable to that observed in other hepaciviruses, such as HCV, where nucleotide sequence differences can reach up to 15% within subtypes [41]. We found no correlation between genetic and geographic distance at large spatial scales, which is likely the result of mutational saturation and evolutionary pressures acting on the NS3 gene (Figure 2) [16,42]. Mutational saturation can lead to an underestimation of genetic divergence among highly divergent sequences, as seen in other RNA viruses, such as PUUV and a related orthohantavirus, Tula virus (TULV) [16,43]. In addition, purifying selection constrains the accumulation of non-synonymous mutations, as indicated by consistently low πa/πs along the fragment of the NS3 gene, reflecting the functional importance of the investigated part of the NS3 protein during viral replication (Figure S2) [24,25,44].

Our phylogenetic analysis of European RHVJ nucleotide sequences revealed local clustering of most sequences as well as the presence of phylogenetically divergent sequences at the same location, resulting in substantial genetic diversity at local scales (Figure 3 and Figure 4A). The overall geographic structuring of the phylogenetic tree is most likely driven by local circulation of RHVJ strains within bank vole populations and limited host movement. However, these factors are unlikely to fully explain the high levels of genetic diversity observed at local scales, as the stability and long-term persistence of viral populations is limited by the short lifespan and strong population fluctuations of bank voles [18]. In other RNA viruses, such as TULV, the presence of phylogenetically divergent sequences at the same location has been associated with contact zones of geographically stable major evolutionary viral lineages [20,45,46]. However, unlike TULV, there is no evidence for distinct evolutionary lineages in RHVJ, and high genetic diversity at local scales seems to be a widespread phenomenon rather than restricted to potential contact zones.

A more likely explanation is that RHVJ dispersal is not limited to short-distance transmission within bank vole populations but also involves occasional long-distance transmission events, through which genetically distinct RHVJ strains are introduced into local host populations. Multiple independent introductions of RHVJ strains into local bank vole populations would increase the genetic diversity at local scales and could lead to the coexistence of closely related and highly divergent RHVJ sequences within the same bank vole population over time, consistent with the phylogenetic patterns observed.

Our phylogenetic trees based on simulated data with occasional long-distance transmission revealed patterns that are comparable to those of RHVJ, characterized by mostly local sequence clusters and monophyletic clustering of sequences from distantly located areas in the simulation space (Figure 4C). Furthermore, most phylogenetic trees resulting from the occasional long-distance transmission scenario had unresolved basal nodes, as also observed in the phylogeny of RHVJ. Given relatively rare long-distance transmission events in our simulations, it is plausible that some phylogenetic trees resembled those generated under strictly local circulation because of stochasticity. Stochastic effects probably contribute to the large variation in diversity within local populations (Figure 3) but it will require much more empirical data to test whether this could also be related to demographic or environmental factors [18]. Overall, our proof-of-principle computer simulations suggest that these phylogenetic patterns are relatively robust and common, but we expect that refined analyses incorporating, e.g., specific information about the ecology of the virus, more populations or full genome data are likely to reveal more details of the transmission dynamics of RHVJ and the robustness of the phylogenetic patterns across the viral genome.

Given that dispersal distances of bank voles are approximately 100–300 m [47], long-distance transmission of RHVJ is unlikely to be driven by vole movement alone and may instead be mediated by vector species transporting RHVJ strains. Hematophagous arthropods such as ticks, mosquitos, flies and fleas are well known vectors that transmit a wide range of pathogens among mammalian hosts [48,49,50,51]. Such vectors could also contribute to the local circulation of viral strains by facilitating short-distance transmission through repeated contact with bank voles of the same host population. In TBEV, for instance, viral circulation mostly involves ticks that transmit the virus to bank voles, as well as other terrestrial animals like roe deer (*Capreolus capreolus*), wild boar (*Sus scrofa*) and red foxes (*Vulpes vulpes*) that carry infected ticks as they move through forest habitats [49,52,53,54]. Phylogenetic analyses of TBEV revealed spatial patterns of genetic variability that are comparable to those of RHVJ, with high genetic diversity at local scales and monophyletic clustering of geographically distant samples in phylogenetic trees [55,56]. Ticks represent a plausible vector species for RHVJ, given their close ecological association with bank voles and their frequent parasitism of this species. As RHVJ has been detected in the blood of bank voles, ticks could acquire the virus during blood feeding and transmit it to other bank voles upon subsequent feeding [9,57]. While ticks are plausible vectors of RHVJ, no RHVJ strain has been reported from ticks or any other arthropod vector to date. However, Collins beach virus, a hepacivirus closely related to rodent-associated hepaciviruses, has been isolated from *Ixodes holocyclus* ticks that were collected from long-nosed bandicoots in Australia [14,58]. The very low viral load detected in these ticks suggests that *I. holocyclus* is unlikely to represent the primary host and may instead function as a potential vector of this virus [14,58].

Long-distance transmission of RHVJ would likely require an additional species that transports infected vectors over larger distances, as seen in TBEV, where migratory birds play a crucial role in spreading infected ticks during seasonal movement [59,60]. In addition, it cannot be excluded that RHVJ also infects other host species that may contribute to viral circulation, although hepaciviruses are generally considered relatively host-specific and current evidence suggests a strong association of RHVJ with bank voles [9,11]. Comparing the codon usage patterns of RHVJ with those of potential arthropod vectors and mammalian hosts, alongside further screening efforts of additional host species, may help evaluate the potential involvement of an arthropod vector and other host species during RHVJ transmission. Identifying potential vector species involved and clarifying the host range of RHVJ are crucial for assessing its potential zoonotic risk, given that transmission via biting arthropods was hypothesized to have played a key role during hepaciviral zoonosis [61].

Including the closely related RHVF in further research may provide comparative insights into the transmission mechanisms of BvHV. Differing modes of transmission between BvHV and HCV would limit their relevance as a model species for HCV research, as viral evolution and host–pathogen interactions strongly depend on the mode of transmission [62]. Pybus et al. suggested that in addition to blood-borne transmission, arthropod-mediated transmission may be involved in the long-term endemic circulation of HCV, based on the inference that endemic HCV strains have been circulating in human populations for centuries before the widespread use of medical injections and blood transfusions [63,64]. However, there is currently no direct evidence for the involvement of a vector species in HCV transmission.

## 5. Conclusions

This study revealed complex spatial patterns of RHVJ sequence diversity and suggests that these are shaped by both local and occasional long-distance transmission of RHVJ mediated by vectors. Continued monitoring of bank vole populations with dense regional sampling is essential to further resolve transmission dynamics. Complementing such efforts by systematic surveys of the viromes of blood-feeding arthropods in and around host populations will help to directly evaluate the role of arthropod vectors in RHVJ transmission. Extending this approach to other hepacivirus species circulating in wildlife and domestic hosts is important for evaluating the risk of spillovers to humans and implementing targeted surveillance and intervention strategies.

## Figures and Tables

**Table 1:** Information on bank voles (*Myodes glareolus*) collected in Switzerland including the ID of each sampling location, sampling years, number of RHVJ RNA-positive individuals and number of sequences that were obtained.

ID	Location	Sampling Years	Bank Voles Positive/Tested	Number of RHVJ Sequences
BiF	Biaufond	2015–2017	8/37	8
BkF	Bütikofen	2015, 2016, 2018	8/22	8
CeG	Cerneux-Godat	2015	0/2	0
ChE	Chalet des Esserts	2020–2023	2/17	2
GrH	Grosshöchstetten	2019	1/12	1
LaC	La Cure	2020–2023	10/22	9 *
LaR	La Racine	2020, 2021	8/22	7 *
LeS	Le Solliat	2017	1/1	1
LeV	Les Verrières	2018, 2019	0/4	0
LoW	Löhrwald	2016	0/2	0
MuT	Mühlethurnen	2018, 2019	3/16	3
PrE	Pré Rodet	2021	4/18	4
RoH	Rothöhe	2015	1/8	1
RoS	Rosshäusern	2016	2/5	2
Total			48/188	46

* Sequencing failed for one RT-PCR-positive sample from this location.

## Data Availability

The source code for the simulations is available at: https://github.com/thomasvongraffenried/RHVJ-Simulations (accessed on 1 May 2026); GenBank accession numbers for RHVJ sequences used in this study are provided in Table S2.

## Supplementary Materials

The following supporting information can be downloaded at: https://www.mdpi.com/article/10.3390/v18060651/s1, Figure S1: Examples of simulation spaces for short-distance transmission (A) and occasional long-distance transmission (B) scenarios of RHVJ spatial sequence evolution (209 generations; seed 10). Green and yellow squares represent the sampling areas where at least 25 sequences each were required for sampling. Dots in these squares represent samples whose sequences were further analyzed, while grey dots outside the square (with no outline) were not sampled. (A) Only short distance transmission, with a short-range kernel of σ = 5 rc. (B) Short and long distance transmission, with 99% of progenies following a short-range kernel of σ = 5 rc and 1% following a long-distance kernel of σ = 200 rc. Rc represents the spatial scale parameter (see text); Figure S2: Sliding window analysis of πa/πs ratio of partial NS3 gene sequences of RHVJ strains from Switzerland (left panel) and all other countries (excluding Switzerland; right panel). Analysis was performed with a window length of 30 and a step size of 10 nucleotides. πa = nucleotide diversity at non-synonymous sites, πs = nucleotide diversity at synonymous sites. As the samples from Switzerland were newly sequenced in this study, a slightly longer sequence (528 nt) could be included in this analysis, compared to the sequences downloaded from GenBank (468 nt); Figure S3: Mantel correlogram illustrating the scale-dependent relationship between genetic and geographic distance of RHVJ strains. Mantel correlation coefficients (r) are shown only for distance classes in which all samples could be included. The horizontal red line indicates r = 0. The correlation for the distance classes 0–200 km was significant (*p* ≤ 0.001; black square at the midpoint of the class), whereas there were no significant correlations for larger distances (white squares); Figure S4: Bayesian phylogenetic tree of all Orthohepacivirus glareoli (RHVJ) sequences based on a 468 nt fragment of the NS3 gene. Novel sequences from Switzerland are marked in red. Posterior probabilities of Bayesian inference values are shown when <1. Nodes with support ≤ 0.8 were removed. Scale bars indicate evolutionary distance in substitutions per nucleotide. Sequence labels contain the three-letter code of the country of origin and the sampling location in italics. A corresponding NS3 gene sequence of Orthohepacivirus myodae (RHVF) was used as outgroup; Table S1: Detailed sampling information of bank voles (*Myodes glareolus*) from Switzerland and estimates of within-population sequence divergence of RHVJ RNA-positive samples. The overall mean nucleotide and amino acid sequence distance for the NS3 gene of RHVJ from Swiss bank voles is included. Mean within-population sequence divergence (p-distance) for nucleotide and amino acid sequences of RHVJ strains were calculated only for locations with more than one viral sequence; Table S2: List of all RHVJ sequences used in this study. Country of origin follows the international three letter codes. N/A: not available.
