# Supplementary material for: Co-Circulation of Divergent Strains Supports Vector-Mediated Transmission of Rodent Hepacivirus J (Orthohepacivirus glareoli)
Source: Viruses. 2026 Jun 5;18(6):651. doi: 10.3390/v18060651 (PMC13307703; doi:10.3390/v18060651)
Supplement: Supplementary file 1 [file viruses-18-00651-s001.zip › viruses-4324425-supplementary.pdf]

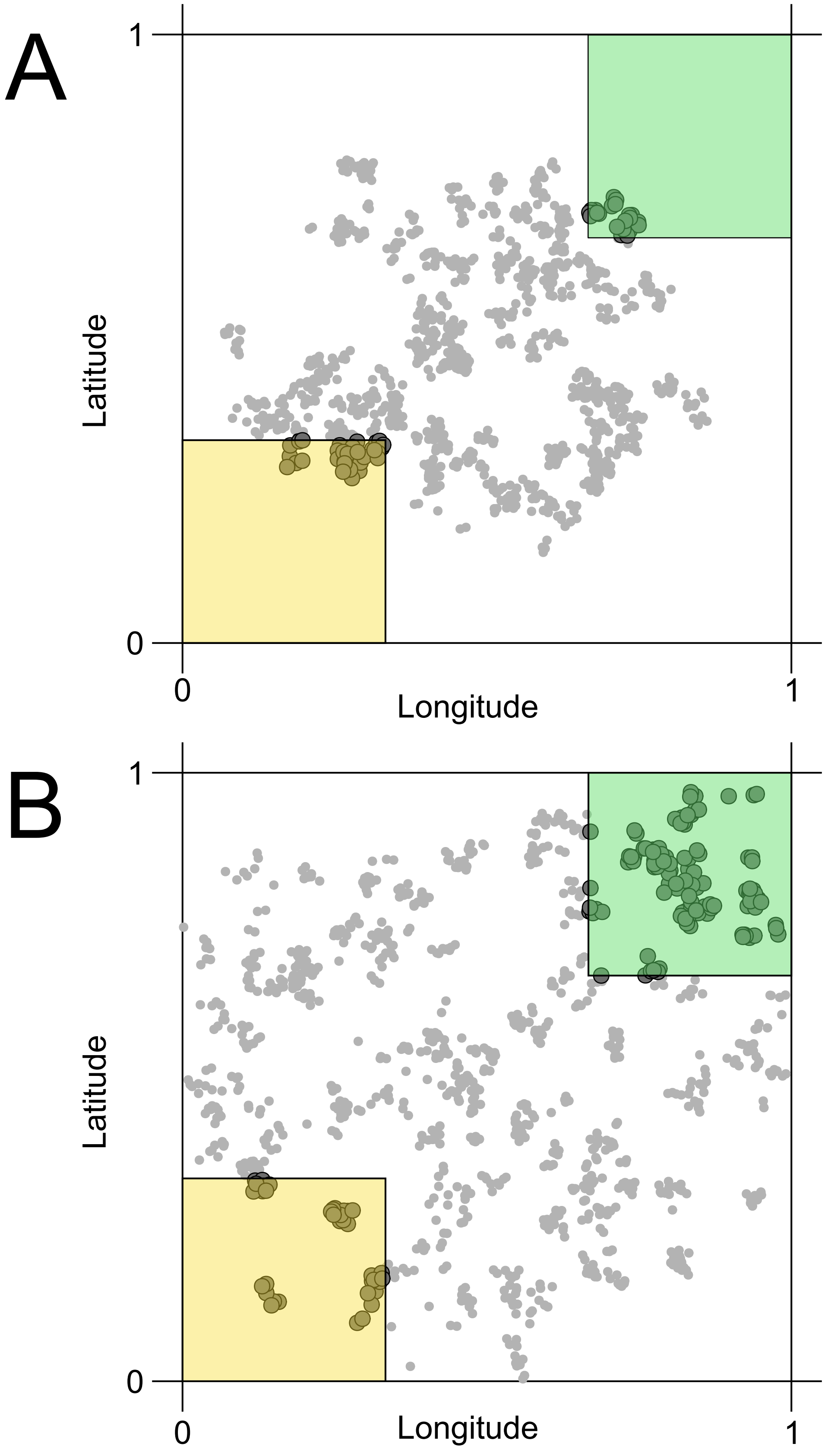

Figure S1: Examples of simulation spaces for short-distance transmission (A) and occasional long-distance transmission (B) scenarios of RHVJ spatial sequence evolution (209 generations; seed 10). Green and yellow squares represent the sampling areas where at least 25 sequences each were required for sampling. Dots in these squares represent samples whose sequences were further analyzed, while grey dots outside the square (with no outline) were not sampled. (A) Only short distance transmission, with a short-range kernel of σ = 5rc. (B) Short and long distance transmission, with 99% of progenies following a short-range kernel of σ = 5rc and 1% following a long-distance kernel of σ = 200rc. Rc represents the spatial scale parameter (see text).

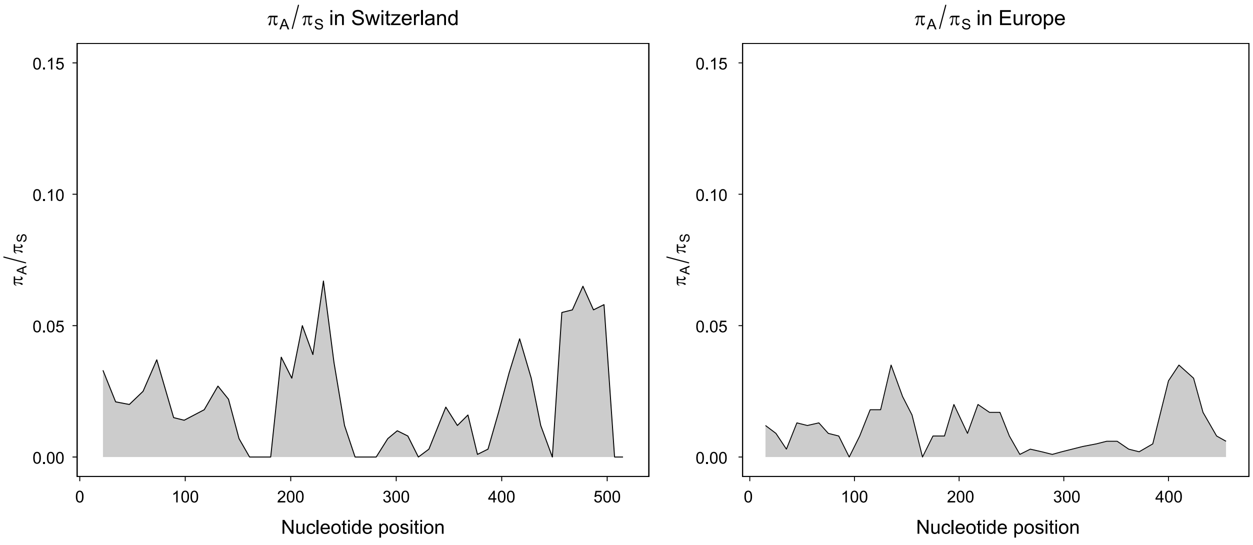

Figure S2: Sliding window analysis of πa/πs ratio of partial NS3 gene sequences of RHVJ strains from Switzerland (left panel) and all other countries (excluding Switzerland; right panel). Analysis was performed with a window length of 30 and a step size of 10 nucleotides. πa = nucleotide diversity at non-synonymous sites, πs = nucleotide diversity at synonymous sites. As the samples from Switzerland were newly sequenced in this study, a slightly longer sequence (528nt) could be included in this analysis, compared to the sequences downloaded from GenBank (468nt).

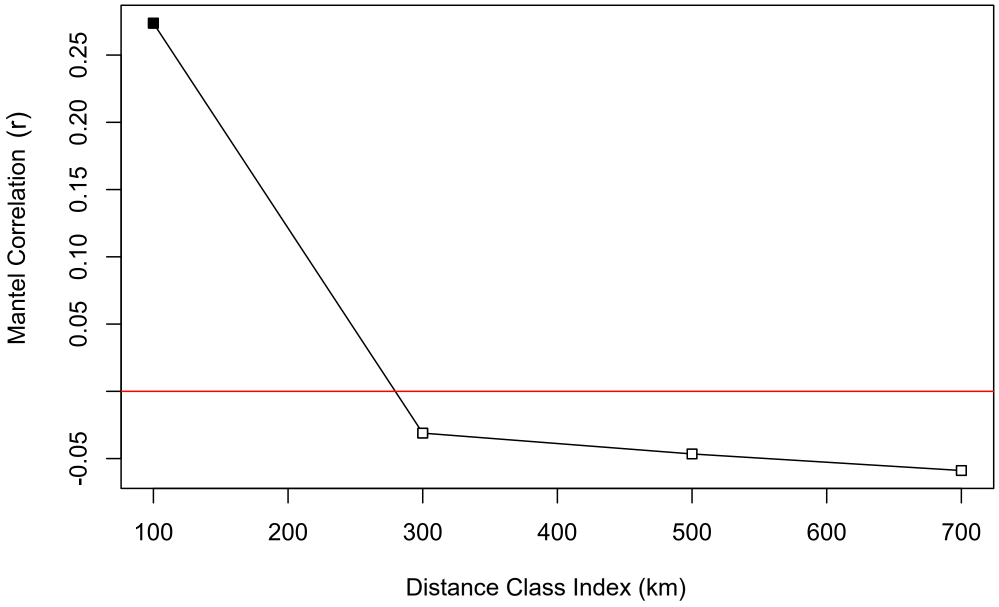

Figure S3: Mantel correlogram illustrating the scale-dependent relationship between genetic and geographic distance of RHVJ strains. Mantel correlation coefficients (r) are shown only for distance classes in which all samples could be included. The horizontal red line indicates r = 0. The correlation for the distance classes 0-200km was significant ($p \leq 0.001$; black square at the midpoint of the class), whereas there were no significant correlations for larger distances (white squares).

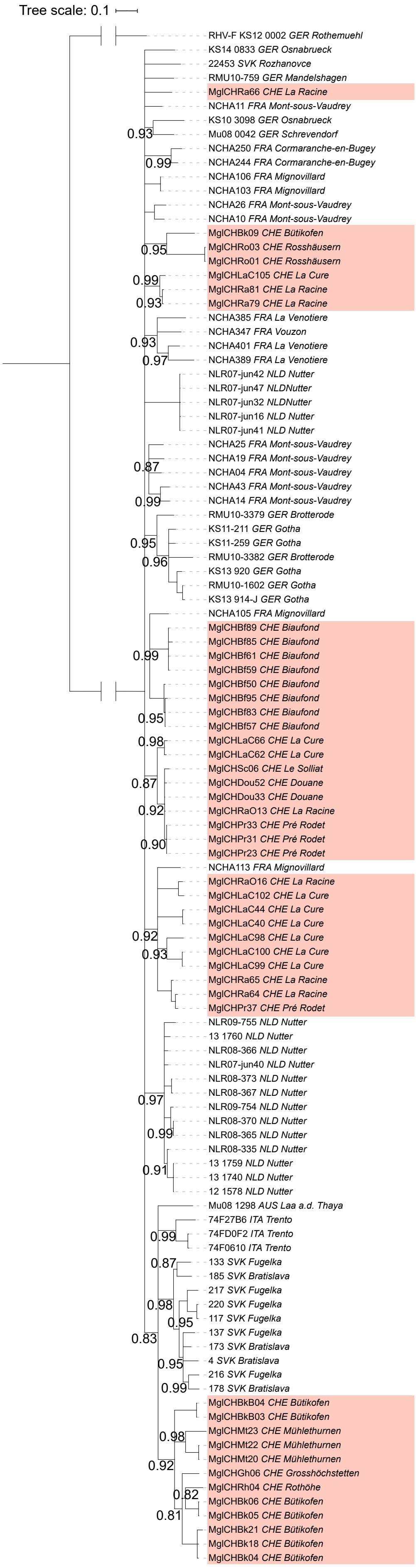

Figure S4: Bayesian phylogenetic tree of all *Orthohepacivirus glareoli* (RHVJ) sequences based on a 468nt fragment of the NS3 gene. Novel sequences from Switzerland are marked in red. Posterior probabilities of Bayesian inference values are shown when < 1. Nodes with support ≤ 0.8 were removed. Scale bars indicate evolutionary distance in substitutions per nucleotide. Sequence labels contain the three-letter code of the country of origin and the sampling location in italics. A corresponding NS3 gene sequence of *Orthohepacivirus myodae* (RHVF) was used as outgroup.

Table S1: Detailed sampling information of bank voles (*Myodes glareolus*) from Switzerland and estimates of within-population sequence divergence of RHVJ RNA-positive samples. The overall mean nucleotide and amino acid sequence distance for the NS3 gene of RHVJ from Swiss bank voles is included. Mean within-population sequence divergence (p-distance) for nucleotide and amino acid sequences of RHVJ strains were calculated only for locations with more than one viral sequence.

ID	Sampling location	Latitude	Longitude	Sampling years	Positive/tested		Number of sequences	Number of nucleotide sequence types	Mean nucleotide sequence distance (%)	Mean amino acid sequence distance (%)
					Per year	Total				
BiF	Biaufond	47.155	6.850	2015<br>2016<br>2017	1/11<br>3/6<br>4/20	8/37	8	5	5.67	1.38
BkF	Bütikofen	47.087	7.627	2015<br>2016<br>2018	6/13<br>2/8<br>0/1	8/22	8	6	10.95	0.07
CeG	Cerneux-Godat	47.176	6.872	2015	0/2	0/2	0	-	-	-
ChE	Chalet des Esserts	46.594	6.189	2020<br>2021<br>2022<br>2023	1/5<br>1/8<br>0/1<br>0/3	2/17	2	2	1.07	0.00
GrH	Grosshöchstetten	46.898	7.633	2019	1/12	1/12	1	1	-	-
LaC	La Cure	46.552	6.162	2020<br>2021<br>2022<br>2023	3/3<br>1/6<br>0/2<br>6/11	10/22	9	7	13.68	1.84
LaR	La Racine	46.583	6.151	2020<br>2021	0/8<br>8/14	8/22	7	7	12.95	2.38
LeS	Le Solliat	46.623	6.230	2017	1/1	1/1	1	1	-	-
LeV	Les Verrières	46.910	6.467	2018<br>2019	0/1<br>0/3	0/4	0	-	-	-
LoW	Löhrwald	46.983	7.409	2016	0/2	0/2	0	-	-	-
MuT	Mühlethurnen	46.806	7.534	2018<br>2019	0/4<br>3/12	3/16	3	2	5.99	0.01
PrE	Pré Rodet	46.562	6.158	2021	4/18	4/18	4	2	8.24	1.94
RoH	Rothöhe	47.044	7.612	2015	1/8	1/8	1	1	-	-
RoS	Rosshäusern	46.930	7.295	2016	2/5	2/5	2	1	0.00	0.00
Total						48/188	46	35	14.83	2.19

Table S2: List of all RHVJ sequences used in this study. Country of origin follows the international three letter codes. N/A: not available

Country	Sequence ID	Accession number	Sampling location	Latitude	Longitude	Year	Sequence	Source
CHE	MglCHLaC40 CHE La Cure	PZ404792	La Cure	46.552	6.162	2020	partial NS3	this study
CHE	MglCHLaC44 CHE La Cure	PZ404793	La Cure	46.552	6.162	2020	partial NS3	this study
CHE	MglCHLaC62 CHE La Cure	PZ404794	La Cure	46.552	6.162	2020	partial NS3	this study
CHE	MglCHLaC66 CHE La Cure	PZ404795	La Cure	46.552	6.162	2021	partial NS3	this study
CHE	MglCHLaC98 CHE La Cure	PZ404796	La Cure	46.552	6.162	2023	partial NS3	this study
CHE	MglCHLaC99 CHE La Cure	PZ404797	La Cure	46.552	6.162	2023	partial NS3	this study
CHE	MglCHLaC100 CHE La Cure	PZ404798	La Cure	46.552	6.162	2023	partial NS3	this study
CHE	MglCHLaC102 CHE La Cure	PZ404799	La Cure	46.552	6.162	2023	partial NS3	this study
CHE	MglCHLaC105 CHE La Cure	PZ404800	La Cure	46.552	6.162	2023	partial NS3	this study
CHE	MglCHPr23 CHE Pré Rodet	PZ404801	Pré Rodet	46.562	6.158	2021	partial NS3	this study
CHE	MglCHPr31 CHE Pré Rodet	PZ404802	Pré Rodet	46.562	6.158	2021	partial NS3	this study
CHE	MglCHPr33 CHE Pré Rodet	PZ404803	Pré Rodet	46.562	6.158	2021	partial NS3	this study
CHE	MglCHPr37 CHE Pré Rodet	PZ404804	Pré Rodet	46.562	6.158	2021	partial NS3	this study
CHE	MglCHRa64 CHE La Racine	PZ404805	La Racine	46.583	6.151	2021	partial NS3	this study
CHE	MglCHRa65 CHE La Racine	PZ404806	La Racine	46.583	6.151	2021	partial NS3	this study
CHE	MglCHRa66 CHE La Racine	PZ404807	La Racine	46.583	6.151	2021	partial NS3	this study
CHE	MglCHRa79 CHE La Racine	PZ404808	La Racine	46.583	6.151	2021	partial NS3	this study
CHE	MglCHRa81 CHE La Racine	PZ404809	La Racine	46.583	6.151	2021	partial NS3	this study
CHE	MglCHRaO16 CHE La Racine	PZ404810	La Racine	46.585	6.133	2021	partial NS3	this study
CHE	MglCHRaO13 CHE La Racine	PZ404811	La Racine	46.585	6.133	2021	partial NS3	this study
CHE	MglCHDou33 CHE Douane	PZ404812	Douane, Chalet des Esserts	46.594	6.189	2020	partial NS3	this study
CHE	MglCHDou52 CHE Douane	PZ404813	Douane, Chalet des Esserts	46.594	6.189	2021	partial NS3	this study
CHE	MglCHSc06 CHE Le Solliat	PZ404814	Le Solliat, Chalet Chez la Tante	46.623	6.230	2017	partial NS3	this study
CHE	MglCHBkB03 CHE Bütikofen	PZ404815	Bütikofen bei Burgdorf	47.045	7.380	2015	partial NS3	this study
CHE	MglCHBkB04 CHE Bütikofen	PZ404816	Bütikofen bei Burgdorf	47.051	7.374	2015	partial NS3	this study
CHE	MglCHBk04 CHE Bütikofen	PZ404817	Bütikofen bei Burgdorf	47.087	7.627	2015	partial NS3	this study
CHE	MglCHBk05 CHE Bütikofen	PZ404818	Bütikofen bei Burgdorf	47.087	7.627	2015	partial NS3	this study
CHE	MglCHBk06 CHE Bütikofen	PZ404819	Bütikofen bei Burgdorf	47.087	7.627	2015	partial NS3	this study
CHE	MglCHBk09 CHE Bütikofen	PZ404820	Bütikofen bei Burgdorf	47.087	7.627	2015	partial NS3	this study
CHE	MglCHBk18 CHE Bütikofen	PZ404821	Bütikofen bei Burgdorf	47.087	7.627	2016	partial NS3	this study
CHE	MglCHBk21 CHE Bütikofen	PZ404822	Bütikofen bei Burgdorf	47.087	7.627	2016	partial NS3	this study
CHE	MglCHGh06 CHE Grosshöchstetten	PZ404823	Grosshöchstetten	46.898	7.633	2019	partial NS3	this study
CHE	MglCHMt20 CHE Mühlethurnen	PZ404824	Mühlethurnen	46.806	7.534	2019	partial NS3	this study
CHE	MglCHMt22 CHE Mühlethurnen	PZ404825	Mühlethurnen	46.806	7.534	2019	partial NS3	this study
CHE	MglCHMt23 CHE Mühlethurnen	PZ404826	Mühlethurnen	46.806	7.534	2019	partial NS3	this study
CHE	MglCHRh04 CHE Rothöhe	PZ404827	Rothöhe, Burgdorf	47.044	7.612	2016	partial NS3	this study
CHE	MglCHRo01 CHE Rosshäusern	PZ404828	Rosshäusern	46.930	7.295	2015	partial NS3	this study
CHE	MglCHRo03 CHE Rosshäusern	PZ404829	Rosshäusern	46.930	7.295	2015	partial NS3	this study
CHE	MglCHBf50 CHE Biaufond	PZ404830	Biaufond	47.155	6.850	2015	partial NS3	this study
CHE	MglCHBf57 CHE Biaufond	PZ404831	Biaufond	47.155	6.850	2016	partial NS3	this study
CHE	MglCHBf59 CHE Biaufond	PZ404832	Biaufond	47.155	6.850	2016	partial NS3	this study
CHE	MglCHBf61 CHE Biaufond	PZ404833	Biaufond	47.155	6.850	2016	partial NS3	this study
CHE	MglCHBf83 CHE Biaufond	PZ404834	Biaufond	47.155	6.850	2017	partial NS3	this study
CHE	MglCHBf85 CHE Biaufond	PZ404835	Biaufond	47.155	6.850	2017	partial NS3	this study
CHE	MglCHBf89 CHE Biaufond	PZ404836	Biaufond	47.155	6.850	2017	partial NS3	this study
CHE	MglCHBf95 CHE Biaufond	PZ404837	Biaufond	47.155	6.850	2017	partial NS3	this study
DEU	RMU10-3382 GER Brotterode	NC_038429	Brotterode	50.826	10.446	2010	whole genome	Drexler et al., (2013)
DEU	KS13 920 GER Gotha	OV121035	Gotha	50.950	10.705	2012	whole genome	Röhrs et al., (2021)
DEU	KS13 914-J GER Gotha	OV121036	Gotha	50.950	10.705	2012	whole genome	Röhrs et al., (2021)
DEU	KS11-211 GER Gotha	KC411799	Gotha	50.950	10.705	2011	partial NS3	Drexler et al., (2013)
DEU	KS11-259 GER Gotha	KC411798	Gotha	50.950	10.705	2011	partial NS3	Drexler et al., (2013)
DEU	RMU10-1602 GER Gotha	KC411779	Gotha	50.950	10.705	2010	partial NS3	Drexler et al., (2013)
DEU	RMU10-3379 GER Brotterode	KC411778	Brotterode	50.826	10.446	2010	partial NS3	Drexler et al., (2013)
DEU	RMU10-759 GER Mandelshagen	KC411776	Mandelshagen	54.123	12.358	2010	partial NS3	Drexler et al., (2013)
DEU	Mu08 0042 GER Schrevendorf	MW822237	Schrevendorf	54.367	10.267	N/A	partial NS3	Schneider et al., (2021)
DEU	KS10 3098 GER Osnabrueck	MW822235	Osnabrück	52.284	8.278	N/A	partial NS3	Schneider et al., (2021)
DEU	KS14 0833 GER Osnabrueck	MW822236	Osnabrück	52.283	8.284	N/A	partial NS3	Schneider et al., (2021)
NLD	NLR08-365 NLD Nutter	KC411796	Nutter	52.423	6.881	2008	whole genome	Drexler et al., (2013)
NLD	NLR08-335 NLD Nutter	KC411797	Nutter	52.423	6.881	2008	partial NS3	Drexler et al., (2013)
NLD	NLR08-366 NLD Nutter	KC411795	Nutter	52.423	6.881	2008	partial NS3	Drexler et al., (2013)
NLD	NLR08-367 NLD Nutter	KC411794	Nutter	52.423	6.881	2008	partial NS3	Drexler et al., (2013)
NLD	NLR08-370 NLD Nutter	KC411793	Nutter	52.423	6.881	2008	partial NS3	Drexler et al., (2013)
NLD	NLR08-373 NLD Nutter	KC411792	Nutter	52.423	6.881	2008	partial NS3	Drexler et al., (2013)
NLD	NLR09-754 NLD Nutter	KC411791	Nutter	52.423	6.881	2009	partial NS3	Drexler et al., (2013)
NLD	NLR09-755 NLD Nutter	KC411790	Nutter	52.423	6.881	2009	partial NS3	Drexler et al., (2013)
NLD	NLR07-jun16 NLD Nutter	KC411789	Nutter	52.423	6.881	2007	partial NS3	Drexler et al., (2013)
NLD	NLR07-jun32 NLDNutter	KC411788	Nutter	52.423	6.881	2007	partial NS3	Drexler et al., (2013)
NLD	NLR07-jun40 NLD Nutter	KC411787	Nutter	52.423	6.881	2007	partial NS3	Drexler et al., (2013)
NLD	NLR07-jun47 NLDNutter	KC411782	Nutter	52.423	6.881	2007	partial NS3	Drexler et al., (2013)
NLD	NLR07-jun42 NLD Nutter	KC411785	Nutter	52.423	6.881	2007	partial NS3	Drexler et al., (2013)
NLD	NLR07-jun41 NLD Nutter	KC411786	Nutter	52.423	6.881	2007	partial NS3	Drexler et al., (2013)
NLD	12 1578 NLD Nutter	MW822266	Nutter	52.423	6.881	N/A	partial NS3	Schneider et al., (2021)
NLD	13 1740 NLD Nutter	MW822267	Nutter	52.423	6.881	N/A	partial NS3	Schneider et al., (2021)
NLD	13 1759 NLD Nutter	MW822268	Nutter	52.423	6.881	N/A	partial NS3	Schneider et al., (2021)
NLD	13 1760 NLD Nutter	MW822269	Nutter	52.423	6.881	N/A	partial NS3	Schneider et al., (2021)
FRA	NCHA04 FRA Mont-sous-Vaudrey	MW822245	Mont-sous-Vaudrey	46.949	5.595	N/A	partial NS3	Schneider et al., (2021)
FRA	NCHA10 FRA Mont-sous-Vaudrey	MW822246	Mont-sous-Vaudrey	46.949	5.595	N/A	partial NS3	Schneider et al., (2021)
FRA	NCHA11 FRA Mont-sous-Vaudrey	MW822247	Mont-sous-Vaudrey	46.949	5.595	N/A	partial NS3	Schneider et al., (2021)
FRA	NCHA14 FRA Mont-sous-Vaudrey	MW822248	Mont-sous-Vaudrey	46.949	5.595	N/A	partial NS3	Schneider et al., (2021)
FRA	NCHA19 FRA Mont-sous-Vaudrey	MW822249	Mont-sous-Vaudrey	46.949	5.595	N/A	partial NS3	Schneider et al., (2021)
FRA	NCHA25 FRA Mont-sous-Vaudrey	MW822250	Mont-sous-Vaudrey	46.951	5.554	N/A	partial NS3	Schneider et al., (2021)
FRA	NCHA26 FRA Mont-sous-Vaudrey	MW822251	Mont-sous-Vaudrey	46.951	5.554	N/A	partial NS3	Schneider et al., (2021)
FRA	NCHA43 FRA Mont-sous-Vaudrey	MW822252	Mont-sous-Vaudrey	46.949	5.595	N/A	partial NS3	Schneider et al., (2021)
FRA	NCHA103 FRA Mignovillard	MW822253	Mignovillard	46.793	6.152	N/A	partial NS3	Schneider et al., (2021)
FRA	NCHA105 FRA Mignovillard	MW822254	Mignovillard	46.793	6.152	N/A	partial NS3	Schneider et al., (2021)
FRA	NCHA106 FRA Mignovillard	MW822255	Mignovillard	46.793	6.152	N/A	partial NS3	Schneider et al., (2021)

FRA	NCHA113 FRA Mignovillard	MW822256	Mignovillard	46.742	6.164	N/A	partial NS3	Schneider et al., (2021)
FRA	NCHA244 FRA Cormaranche-en-Bugey	MW822257	Cormaranche-en-Bugey	45.941	5.628	N/A	partial NS3	Schneider et al., (2021)
FRA	NCHA250 FRA Cormaranche-en-Bugey	MW822258	Cormaranche-en-Bugey	45.925	5.614	N/A	partial NS3	Schneider et al., (2021)
FRA	NCHA347 FRA Vouzon	MW822259	Vouzon	47.661	2.117	N/A	partial NS3	Schneider et al., (2021)
FRA	NCHA385 FRA La Venotiere	MW822260	La Venotière	47.740	1.769	N/A	partial NS3	Schneider et al., (2021)
FRA	NCHA389 FRA La Venotiere	MW822261	La Venotière	47.740	1.769	N/A	partial NS3	Schneider et al., (2021)
FRA	NCHA401 FRA La Venotiere	MW822262	La Venotière	47.740	1.769	N/A	partial NS3	Schneider et al., (2021)
ITA	74F0610 ITA Trento	MW822263	Trento	46.022	10.907	N/A	partial NS3	Schneider et al., (2021)
ITA	74F27B6 ITA Trento	MW822264	Trento	46.022	10.907	N/A	partial NS3	Schneider et al., (2021)
ITA	74FD0F2 ITA Trento	MW822265	Trento	46.022	10.907	N/A	partial NS3	Schneider et al., (2021)
AUT	Mu08 1298 AUS Laa a.d. Thaya	MW822270	Laa an der Thaya	48.736	16.345	N/A	partial NS3	Schneider et al., (2021)
SVK	4 SVK Bratislava	MW822271	Bratislava	48.190	17.080	N/A	partial NS3	Schneider et al., (2021)
SVK	178 SVK Bratislava	MW822273	Bratislava	48.200	17.100	N/A	partial NS3	Schneider et al., (2021)
SVK	185 SVK Bratislava	MW822274	Bratislava	48.200	17.100	N/A	partial NS3	Schneider et al., (2021)
SVK	173 SVK Bratislava	MW822275	Bratislava	48.190	17.080	N/A	partial NS3	Schneider et al., (2021)
SVK	117 SVK Fugelka	MW822276	Fugelka	48.380	17.300	N/A	partial NS3	Schneider et al., (2021)
SVK	133 SVK Fugelka	MW822277	Fugelka	48.380	17.310	N/A	partial NS3	Schneider et al., (2021)
SVK	137 SVK Fugelka	MW822278	Fugelka	48.380	17.310	N/A	partial NS3	Schneider et al., (2021)
SVK	216 SVK Fugelka	MW822279	Fugelka	48.380	17.300	N/A	partial NS3	Schneider et al., (2021)
SVK	217 SVK Fugelka	MW822280	Fugelka	48.380	17.300	N/A	partial NS3	Schneider et al., (2021)
SVK	220 SVK Fugelka	MW822281	Fugelka	48.380	17.300	N/A	partial NS3	Schneider et al., (2021)
SVK	22453 SVK Rozhanovce	MW822282	Rozhanovce	48.760	21.358	N/A	partial NS3	Schneider et al., (2021)
